# Differentiation between two strains of microalga *Parachlorella kessleri* using modern spectroscopic method

**DOI:** 10.1186/s40529-014-0053-7

**Published:** 2014-07-12

**Authors:** Marwa M Saleh, Dmitry N Matorin, Bolatkhan K Zayadan, Daria A Todorenko, Evgenii P Lukashov, Mona M Gaballah

**Affiliations:** 1 Department of Biotechnology, Al-Farabi Kazakh National University, 71, Al-Farabi, ave, Almaty, 050040 Kazakhstan; 2 Faculty of Biology, Moscow State University, Vorobyevi Gory, 119992 Moscow Russia; 3 Botany Department, Faculty of Science, Suez Canal University, Ismaillia, 41522 Egypt

**Keywords:** Parachlorella kessleri, Chlorophyll a fluorescence, Delayed fluorescence, JIP-test, Photosynthesis

## Abstract

**Background:**

The differentiation between wild type of *Parachlorella kessleri* and its mutant strains *PC Mut2*, *PC Mut4* by using the Multi-functional Plant Efficiency Analyzer (М-РЕА-2) was studied. Mutant algal cells of *P. kessleri* have been obtained by UV-C during 3 and 10 min respectively.

**Results:**

Light-induced kinetics of prompt fluorescence (OJIP transients), delayed fluorescence and modulated reflection at 820 nm (redox transitions of P_700_ in PSI) showed disturbance of electron transport flow in photosystem II (PSII) and an increase fraction of non-reducing centers of secondary quinone acceptors of electron (Q_B_). In addition, the amplitudes of the fast and slow peak in the kinetics of the delayed light emission and non-photochemical fluorescence quenching *(* NPQ) were significantly reduced in mutant cells, indicating low level of the membrane energization of photosynthetic membranes. Changes of photosynthetic reactions of mutants may lead to an increase of the carotenoids content, which protect cells against the light stress.

**Conclusion:**

It is suggested to use parameters of induction curves of prompt and delayed fluorescence to characterize mutant algal cells in biotechnological studies.

**Electronic supplementary material:**

The online version of this article (doi:10.1186/s40529-014-0053-7) contains supplementary material, which is available to authorized users.

## Background

The solar radiation sustains life on Earth. However significant increase of UV-C radiation may lead to inhibition of many biological processes. The major cellular targets of UV-C are various biomolecules, which directly absorb this radiation or are indirectly affected by various UV-C induced photochemical reactions. The biological and, ultimately, ecological consequences are numerous (Charles [2008]). It is known that UV-C induces some physiological effects such as declining photosynthetic rates, which can be related not only to damaged biomolecules, but also to ultrastructural changes in organelles or membranes (Holzinger and Lütz [2006]). Typical alterations include swollen mitochondrial cristae, disrupted thylakoids or detached phycobilisomes in chloroplasts, bent-shaped dictyosomes, and damaged plasmalemma. An intact ultrastructure of the algal cell is a prerequisite for the optimum functioning of all physiological processes (Charles [2008]). *Parachlorella kessleri* was described as a new genus, derived from the true spherical Chlorella. The wild strain SAG 211-11 h was formerly referred to as *Chlorella vulgaris* but later reassigned as *Chlorella kessleri* (Fott and Nováková [1969]). Today this species is referred to as *P. kessleri* (Krienitz et al. [2004]).

Fluorescence methods are used in biotechnological experiments for monitoring photosynthesis processes, which give detailed information of primary defects of cell metabolism, mainly at the membrane level (Schreiber [2004]; Antal et al. [2009]; Matorin et al. [2013]; Schansker et al. [2014]). The basis of fluorescence methods lies in the ability of chlorophyll located in photosynthesis membranes to serve as a natural detector for the algae cells, emitting quanta fluorescence. Importantly, such methods allow to gain information of alga state in real time. Measurement of the ratio between the fluorescence intensity under the photosynthesis saturating illumination (*F*_*M*_) and under low light intensity, which induces no changes in the state of the photosynthetic apparatus (*F*_*O*_) makes it possible to determine the maximum efficiency of the PSII processes, which is equal to *F*_*V*_/*F*_*M*=_ (*F*_*M*_ - *F*_*O*_)/*F*_*M*_. The *F*_*V*_/*F*_*M*_ ratio presents dimensionless characteristics of the efficiency of photosynthesis, which is independent of the species of organisms. Measurements of fluorescence induction curves are carried out during several seconds by using PAM or PEA instrument. Measurements of fluorescence induction curves with high resolution (starting from 20 μs) have been recently used in studies of photosynthetic reactions in higher plants and algae cultures (Schreiber [2004]; Strasser et al. [2005]; Matorin and Rubin [2012]). The M-PEA-2 instrument allows to measure separate reactions in PSI and PSII simultaneously (Strasser et al. [2010]; Oukarroum et al. [2013]; Bulychev et al. [2013]). Moreover, it monitors induction changes of delayed fluorescence, which indicate the level of the membrane energization (Goltsev et al. [2009]).

In the present paper we investigated processes in PSI and PSII of wild type of *P. kessleri* and two mutant strains by using a newly designed Multi-functional Plant Efficiency Analyzer (M-PEA-2, Hansatech). This instrument gives a comprehensive picture of primary photosynthetic events by simultaneous recording the prompt and delayed fluorescence kinetics and reflectance at 820 nm at high time resolution. Here we introduce results of the first attempt of assessment of photosynthetic characteristics of the mutant strains of algal cells by using M-PEA-2.

## Methods

### Strains and growth conditions

The strain of *Parachlorella kessleri* microalga was obtained from Al-Farabi Kazakh National University, Biotechnology Department culture collection. *P. kessleri* wild type strain was grown in tris-acetate-phosphate (TAP) medium (pH 6–7) (Gorman [1965]), in 250 ml Erlenmeyer flask at 28°C under continuous illumination of photosynthetic photon flux density (PPFD) 120 μmol photons m^−2^ s^−1^ and constant shaking.

### UV irradiation and mutagenesis

According to Harris ([1989]), 5 ml of the liquid culture with a density of 1×10^6^/ml algal cells were placed in 9 cm Petri dish, where the culture formed a thin layer, covering the bottom. The dish was placed on shaker at 20 rpm and exposed to UV-C lamp (254 nm and 40 erg/mm^2^) at distance 15 cm during 3 and 10 min respectively. After UV irradiation, the cells were inoculated in solid TAP medium and incubated in darkness for 24 h to prevent photoreactivation. After the 24 h, some dishes were incubated photoautotrophically (photon flux density 120 μmol photons m^−2^ s^−1^), others were incubated heterotrophically (in dark) during 15 days.

### Selection of the mutant strains

After the incubation period, two mutant strains were selected based on their phenotypic characteristics, using colonies whose color and size differed from the wild type colonies. These mutant strains, *PC Mut2* and *PC Mut4*, were obtained from *P. kessleri* after irradiation time of 3 and 10 min respectively. These mutants were transferred from the solid to liquid medium and kept under phototrophic growth conditions (photon flux density 120 μmol photons m^−2^ s^−1^).

### Analysis of chlorophyll *a* and *b* content

Spectrophotometry method was used according to Merchant et al. ([2007]). The calculation of the concentration of the pigments was determined by the optical density of pigment solutions at appropriate wavelength.

### Chlorophyll fluorescence and absorbance measurements

Light-induced kinetics of prompt Chl fluorescence (PF), delayed Chl fluorescence (DF) and modulated reflectance at 820 nm (MR) were recorded by using Multi-functional Plant Efficiency Analyzer (M-PEA-2, Hansatech Instrument Ltd., King’s Lynn, Norfolk, UK). Prior to the measurements, the cells were concentrated on a membrane filter. Then a thin layer of cells was fixed by a special clip on the surface of the measuring chamber and incubated in the darkness for 10 min. It has previously been verified in algal suspension by using Aqua-Pen fluorometer (Photon System Instruments, Czech Republic) that concentration algal cells on the filter surface doesn’t affect the physiological state of cells (Matorin et al. [2013]).

The design of the measuring chamber, timing protocol of a single measurement, and data acquisition procedure for M-PEA-2 can be found in Strasser et al. ([2010]). PPFD and duration of actinic illumination were set to 1300 μmol photons m^−2^ s^−1^ and 3 s, respectively. In the M-PEA-2, the DF induction curves are constructed from the DF intensities at certain delay time-point in the kinetics of DF decay recorded during the dark interruptions of the actinic light, which induces OJIP (intermediate levels of the light-induced fluorescence curve) rise. In our experiments, the delay time point in the DF decay curve was set to 50 μs. It is assumed that submillisecond DF components are generated from PSII reaction centers in the state Z^+^P_680_Q_A_^−^ (Goltsev et al. [2009]), where Z is a tyrosine Z residue, P_680_ is a primary electron donor, and Q_A_ is a primary quinone acceptor in PSII.

Fluorescence was measured by using Water-PAM fluorometer (Heiz Walz, Effeltrich, Germany). Ratio *F*_*V*_/*F*_*M*_ is the maximum quantum yield of PSII photochemistry was measured in dark-adapted sample, where *F*_*V*_ *= F*_*M*_*-F*_*O*_*.* Measurements in the light were performed with progressive increase of light intensity from 0 to 800 μmol photons m^−2^ s^−1^. At the end of each illumination session, *F*_*M*_*’* and fluorescence yield in light *F(t)* were measured using saturating flash (0.8 s, 3000 μmol photons m^−2^ s^−1^) (Schreiber [2004]; Herlory et al. [2007]). These parameters were used to calculate non-photochemical quenching of chlorophyll fluorescence *NPQ = (F*_*M*_*- F*_*M*_*’)/F*_*M*_', the quantum yield of photochemical conversion of light energy in PSII, as *Y = (F*_*M*_*’- F(t))/F*_*M*_', and the relative yield of noncyclic electron transport at given light intensity *rETR = Y* × *E*_*i*_ *× 0.5*, where *E*_*i*_ is illumination intensity (μmol photons m^−2^ s^−1^) (Lippemeier et al. [1999]). Parameters are designated according to generally accepted nomenclature (Schreiber [2004]).

Light absorption spectrum of samples was measured by using HITACHI-557 spectrophotometer. Fluorescence spectra, excitation spectra, and fluorescence decay times in algae were measured by using Fluorolog-3 (Horiba Jobin Yvon) spectrofluorometer with the TCSPC (time correlated single photon counting) option (O’Connor and Philips [1984]). Fluorescence was measured at 685 nm under excitation with pulse LED at 390 nm. Fluorescence decay kinetics was approximated with the sum of two exponential curves.

All measurements were repeated no less than five times. The figures present data of three replicate experiments at least.

## Results and discussion

In this study mutations were induced by UV-C radiation. UV-C mutagenesis offers many advantages such as less pollution, simple operation, and sterile cultivation conditions. After UV-C irradiation, some colonies appeared with phenotypic characteristics that differed in color and size from the control colonies. The colonies were selected based on their phenotypic appearance, transferred into liquid medium and then subcloned several times to insure that the mutation was stable and no changes in their phenotypic characteristics occurred. The major cellular targets of UV-C are different biomolecules, which directly absorb this radiation, or which are indirectly affected by various UV-C induced photochemical reactions (Charles [2008]). In the present paper two mutant strains of *P. kessleri* after irradiation during 3 and 10 min respectively were selected and designated as *PC Mut2* and *PC Mut4*. It was shown microscopically that mutant cells are smaller, than wild type cells. All strains were grown in the liquid TAP medium under phototrophic conditions. The algal cultures of the wild and mutant strains were different in color.

**Figure 1:**
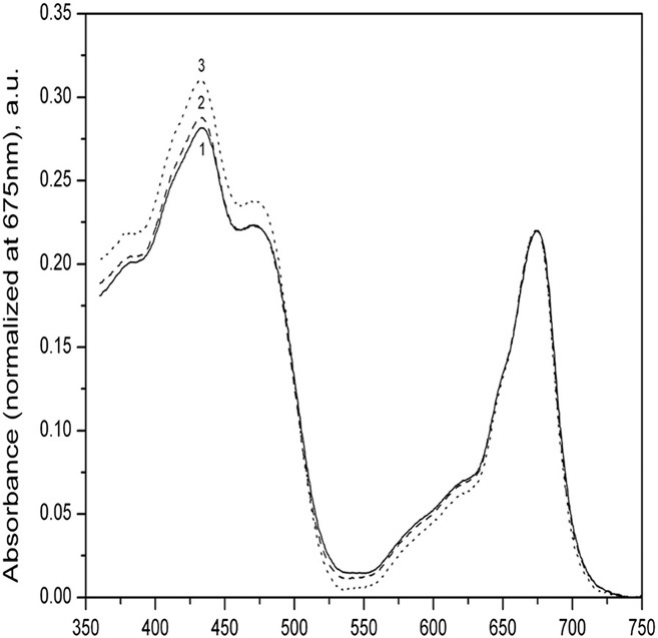
**Absorption spectra of suspensions.** wildtype (curve 1) and mutant strains *PCMut2* (curve 2) and *PCMut4* (curve 4). The spectra are normalized to absorption in the red maximum of chlorophyllа *а* at 675 nm.

**Figure 2:**
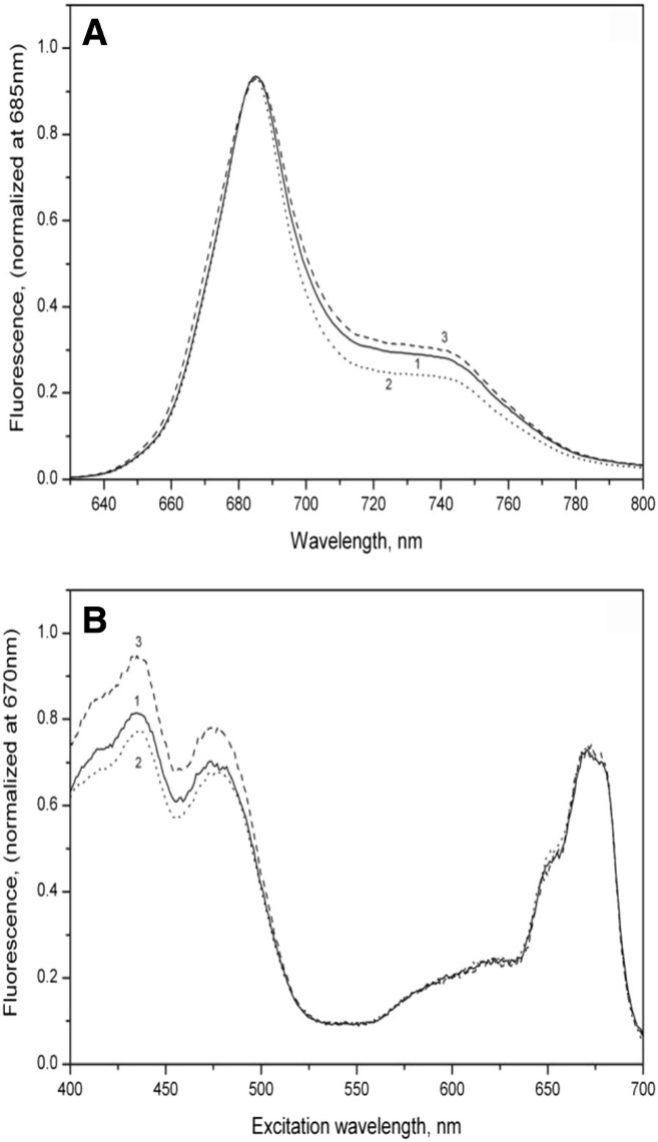
**Fluorescence spectra. (А)** under excitation at 430 nm **(В)** at 720 nm in wildtype (curve 1) and mutant strains *Parachlorella kessleri* cells (*PCMut2*- curve 2) and (*PCMut4* -curve 3). Fluorescence spectra are normalized to the maximum fluorescence values at 690 nm. Fluorescence excitation spectra are normalized to corresponding values of the red absorption maximum at 680 nm.

Absorption spectra of wild type and mutant strains of cells are in Figure 1. The spectra have been normalized to the maximum chlorophyll absorption in red region at 675 nm. Relative increase of carotenoid absorption at the Soret band was noted in mutant strains of *P. kessleri*. The highest increase was observed in *PC Mut4*. This was confirmed by measurements of fluorescence excitation spectra (Figure 2В) The se spectra showed an increase in the region of carotenoid absorption. Fluorescence spectra did not differ significantly (Figure 2А), which may be related to the fact that carotenoids in algae do not fluorescence, but efficiently transfer excitation energy of chlorophyll *a*, which is fluorescence emitter.

**Figure 3:**
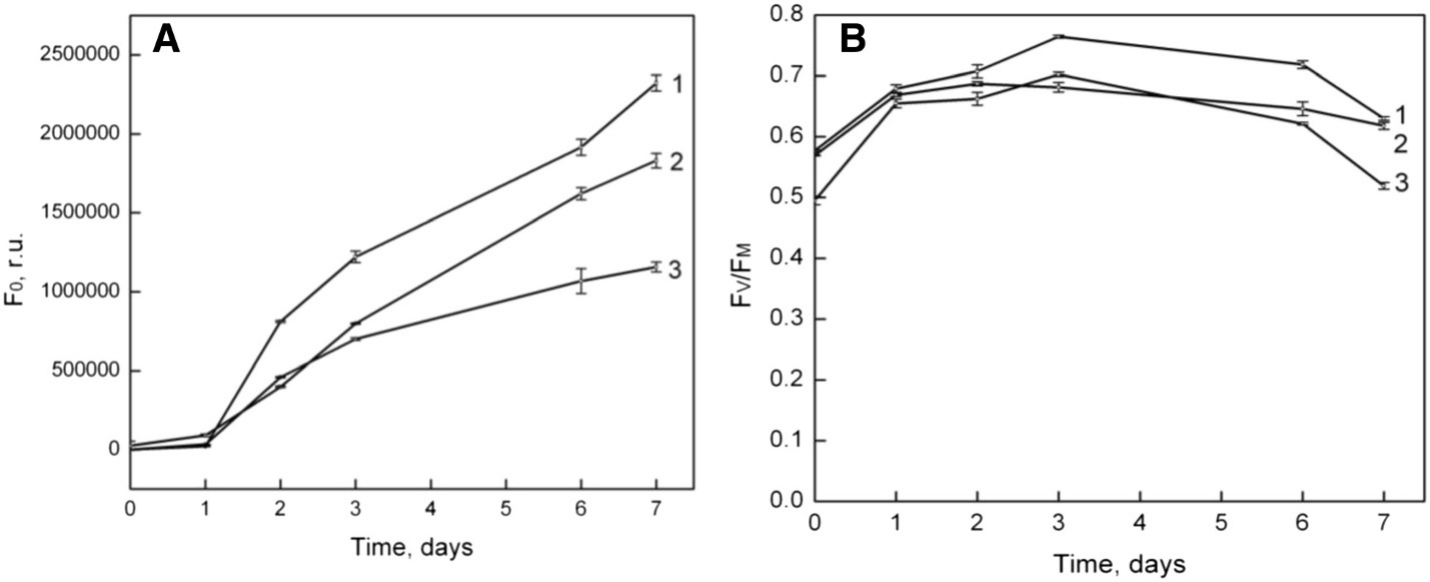
**Changes in fluorescence parameters. (A)** *F*_*O*_**(B)** *F*_*V*_*/F*_*M*_ in suspension wildtype (curve 1) and mutant strains of *Parachlorella kessleri* cells *PCMut2* (curve 2) and *PCMut4* (curve 3) during growth. The graphs are plotted as means ± SEM.

It is known that the constant fluorescence level *F*_*O*_ highly correlates with the total content of pigments in the photosynthetic apparatus of algal cells, that harvest light energy, and *F*_*O*_ level correlates with cell number (Matorin et al. [2004]). The ratio *F*_*V*_/*F*_*M*_ reflects the maximum quantum yield in PSII. This value is related to the processes of water splitting and oxygen evolution in PSII. Changes of fluorescence parameters *(F*_*O*_, *F*_*V*_/*F*_*M*_) are shown in Figure 3.

Figure 3 shows fast increase of algal cells of wild type (curve 1) after the lag-phase (1^st^ day), as measured by *F*_*O*_. *PC Mut2* (curve 2) and *PC Mut4* (curve 3) mutants demonstrate a lower growth rate during investigation period. Photosynthetic activity (*F*_*V*_/*F*_*M*_ ratio) of wild type cells slightly increased during growth, reaching the maximum value at the 3^rd^ day. After prolonged culturing (6^th^ day) photosynthetic activity, judged by *F*_*V*_/*F*_*M*_ ratio, was decreased, possibly due to exhaustion of mineral nutrients in the growth medium. Mutants *PC Mut2* and *PC Mut4* had a lower *F*_*V*_/*F*_*M*_ values.

**Figure 4:**
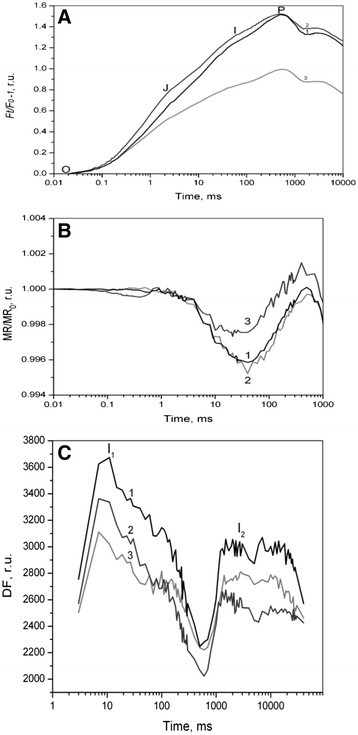
**Light-induced kinetics. (A)** of PF (OJIP), **(B)** MR, **(C)** DF. After turning on the light in the culture of wildtype and mutant strains of *Parachlorella kessleri* cells. Light intensity was 1300 μE/(m^2^ s). Kinetic curves were simultaneously recorded with M-PEA instrument. OJIP and MR curves are normalized to the initial values at 20 ns and represented as *F(t)/F*_*M*_ – 1 and MR/MR_0_ ratios, respectively. Each curve represents an average of 3 replicate experiments.

For more detailed investigation of photosynthetic activity of wild type and two mutant strains *P. kessleri* cells, fluorescence parameters were measured by using M-PEA-2 (Figure 4). M-PEA-2 provides extended possibilities to evaluate the functional state of PSII and PSI by measuring light-induced kinetics of the PF (OJIP transients), DF, and MR with a high resolution (Strasser et al. [2010]; Oukarroum et al. [2013]). Although there is no full consensus on the origin of the complex OJIP rise kinetics, it is commonly accepted that the underlying mechanisms may involve successive decrease in photochemical and non-photochemical fluorescence quenching due to the light-induced PSII closure and PQs reduction (Antal et al. [2009]; Strasser et al. [2010]). PSII closure refers to reduction of the primary quinone acceptor, Q_A_, in PSII.

Figure 4 presents kinetic curves of fluorescence induction after switching the light on, normalized against the O level. In the control cells, the shape of the fluorescence curve corresponded to that described in the literature (Lazár [2009]; Strasser et al. [2010]). Usually, several components are observed in the kinetics of fluorescence induction in response to intense illumination, namely, the O-J-I-P transitions. The initial level O corresponds to chlorophyll fluorescence intensity with open RCs of PSII (*F*_*O*_) when all Q_A_ are oxidized. The time period required to reach this level is up to 20 μs. The O-J phase is caused by light induced reduction of Q_A_, while the subsequent phases reflect mainly its further accumulation in reduced state, caused by a decrease in its reoxidation rate as a result of reduction of Q_B_ acceptors and the pool of quinone. The shape of the O-J-I-P curve was changed in the mutants (Figure 4). The mutant *PC Mut4* showed marked decrease in the contribution of the photochemical phase J-I-P, which indicates interruption of the electron flow from PSII to plastoquinone pool. To quantitatively analyze the characteristics of the primary processes of photosynthesis on the basis of O-J-I-P kinetic curve parameters, the so called JIP test (Strasser et al. [2010]) was used. The JIP test uses the following parameters of the fluorescence induction kinetic curve: (a) fluorescence intensity at 20 μs (*F*_*O*_), 300 μs (F300 μs), 2 ms (*F*_*J*_), 30 ms (*F*_*I*_), 6 s (*F*_*6s*_), and *F*_*P*_ (*F*_*M*_, the maximum fluorescence yield); (b) time to reach the maximum fluorescence (*t F*_*M*_), and (с) area under the kinetic curve below the *F*_*M*_ level. These characteristics were used to calculate the following parameters presented in (Table 1): (1) maximum efficiency of PSII (*F*_*V*_/*F*_*M*=_ (*F*_*M*_ - *F*_*O*_)/*F*_*M*_); (2) relative amplitude of the O–J phase (*V*_*J*_ = (*F*_*J*_ – *F*_*O*_)/*F*_*V*_, which reflects the fraction of non Q_B_-reducing RC of PSII, which lack the contact between the two consecutive PSII acceptors, Q_A_ and Q_B_; (3) relative amplitude of the J–I phase (*V*_I_ = (*F*_I_ – *F*_J_)/*F*_*V*_): (4) *M*_O_ parameter (*M*_O_ = 4 × (*F*_300μs_ – *F*_*O*_)/ *F*_*V*_), which reflects the initial slope of the induction curve; *М*_O_ value is proportional to the rate of Q_A_ reduction under conditions when Q_B_ and the pool of plastoquinones are mainly in the oxidized state; (5) *S*_*M*_ = (Area)/*F*_*V*_, normalized value of *F*_*V*_, the area between the OJIP curve and *F*_*M*_ value, which reflects the total number of PSII turnover during the OJIP phase of growth of the fluorescence yield; (6) *ABS/RC = M*_*O*_*(1/F*_*M*_*) (l/V*_*J*_*)*, average value of absorbed photon streams in RC of PSII (observed size of the active PSII antenna); (7) capacity for pH induced non-photochemical fluorescence quenching (*q*_E_ = (*F*_*M*_ – *F*_*6s*_)/*F*_*V*_); and (8), capability of the quinone pool to quench fluorescence *q*_PQ_ = (*F*_*M*_ – *F*_*I*_)/*F*_*V*_. Many parameters of the JIP-test are mutually dependent; that is, change in some of them leads to changes in the others. Independent parameters (*V*_J_ and *М*_O_) derived form the analysis of the O–J phase provide the information on Q_A_ reduction. From the *V*_I_ and *S*_M_ parameters, it is possible to derive information on further accumulation of reduced which occurs due to reduction of Q_B_ and the quinone pool.

**Table 1:** **Parameters of OJIP kinetics of fluorescence induction curve of wild type and three mutant strains** ***Parachlorella kessleri*** **cells**

JIP-test parameters		Wild typePC*	PC Mut2*	PC Mut4*
*F*_*V*_ */F*_*M*_	Maximum quantum yield of charge separation in PS2	0.610 ± 0.01	0.607 ± 0.01	0.506 ± 0.01
*V*_*J*_	Relative amplitude of the O-J phase	0.38 ± 0.1	0.47 ± 0.01	0.47 ± 0.003
*V*_*I*_	Relative amplitude of the J-I phase	0.96 ± 0.02	0.96 ± 0.01	0.94 ± 0.004
*Mo*	Initial slope of the O-J fluorescence phase	0.60 ± 0.04	0.73 ± 0.02	0.87 ± 0.03
*S*_*M*_	Area between the fluorescence kinetic curve (O–J–I–P) and the level of Fm normalized against the Fv value	40.97 ± 4.24	38.72 ± 2.32	30.06 ± 1.01
*ABS/RC*	Average value of absorbed photon flows in PS2 RC (of apparent size of the active antenna in PS2)	2.57 ± 0.2	2.85 ± 0.1	3.51 ± 0.2
*qE*	Capacity for pH-induced non-photochemical fluorescence quenching	0.14 ± 0.01	0.12 ± 0.01	0.10 ± 0.01
*qPQ*	Capacity of the quinone pool for fluorescence quenching	0.24 ± 0.04	0.20 ± 0.01	0.19 ± 0.02

*Figures are expressed as mean ± SD.

The kinetic parameters of fluorescence induction were recorded with M-PEA-2 instrument under actinic illumination of 1300 μmol photons m^−2^ s^-1^.

It is assumed that the O–J phase of fluorescence induction reflects the accumulation of both reducing and non-reducing PSII (Antal et al. [2009]; Strasser et al. [2010]). The hypothesis is supported by the fact that DCMU (3-(3,4-dichlorophenyl)-1,1-dimethylurea), which inhibits electron transport between Q_A_ and Q_B_, leads to fast growth of fluorescence to the maximum level within 2 ms, corresponding to the appearance of the J peak in the control. Analysis demonstrated that, in cells of the strains obtained, the number of non-Q_B_-reducing centers of PSII, which are incapable of the quinone pool reduction, was increased. This was supported by a number of other parameters (Table 1). Accordingly, values of the parameter *М*_*O*_, which reflects the initial slope of the induction curve, increased. Total flow of photons, absorbed by the pigments of the PSII antenna and normalized against the RC number (*ABS/RC*), increased in the cultures of mutant strains *P. kessleri*, as compared to the control values. At the same time, the relative amplitude of the J–I phase (V_I_) was practically unchanged.

Prompt fluorescence induction curves also demonstrated the suppression of fluorescence decay after reaching the maximum due to ΔpH dependent nonphotochemical quenching (*q*_E_ = (*F*_*M*_ – *F*_*6s*_)/*F*_*V*_)). The value of this parameter decreased in mutant strains, which evidences a decrease in the membrane energization. The ability of the quinone pool to quench fluorescence *q*_PQ_ = (*F*_*M*_ – *F*_*I*_)/*F*_*V*_ in algae of mutant strains was also reduced.

**Figure 5:**
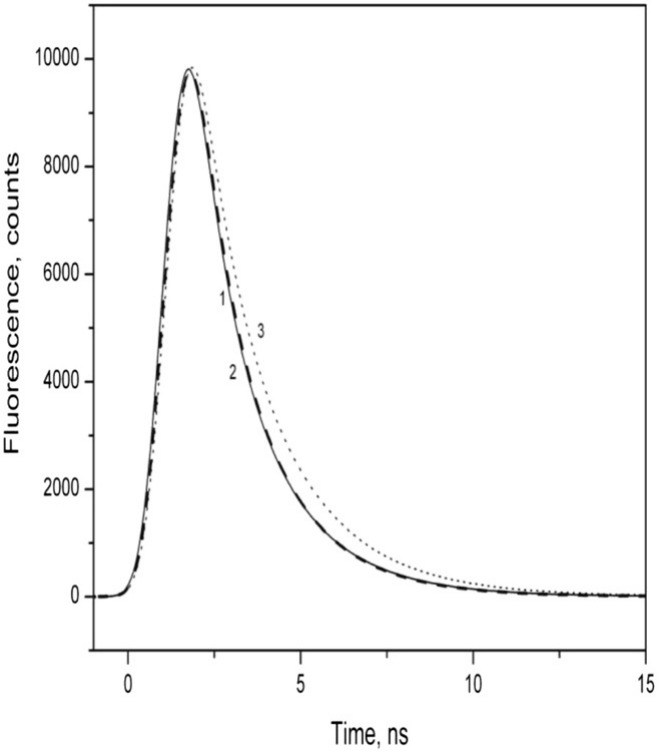
**Kinetics of fluorescence decay.** In wildtype (curve 1) and mutant strains of *Parachlorella kessleri* cells (*PCMut2*- curve 2) and (*PCMut4* -curve 3). The measurements were performed with a Fluorolog-3 spectrofluorometer at 685 nm, using TCSPC method with sample activation with light at 1 Mhz frequency with a LED 390 nm by pulses with half-widths of 0.9 ns.

Figure 5 shows the kinetics of fluorescence decay in the wild type and mutant strains of *P. kessleri* cells. Bi-exponential fitting of decay kinetics was applied to distinguish two components, one of them is related to energy trapping (fast component) and the other to charge stabilization and recombination in PSII reaction centers (slow сcomponent) (Volgusheva et al. [2007].).

In our experiments we found a fast component with half-time about 1 ns and a slower component with half-time more than 2 ns (Table 2). In the wild type and *PC Mut2*, the ratio of these components was 40 and 60%, respectively. In *PC Mut4*, the half-time of the slow component increased from 2.2 to 2.5 ns, and its relative magnitude also increased to 72%.

**Table 2:** **Changes of amplitude and duration of fast (A1, τ**_**1**_ **) and slow (A2, τ**_**2**_ **) components of fluorescence decay kinetics of wild type and mutant strains** ***Parachlorella kessleri*** **cells**

Samples	A1 (%)	A2 (%)	τ_1_(ns)*	τ_2_(ns)*
Wild type	40	60	1.0 ± 0.13	2.24 ± 0.03
*PC Mut 2*	37	63	0.97 ± 0.14	2.18 ± 0.03
*PC Mut 4*	28	72	1.09 ± 0.23	2.47 ± 0.04

*Figures are expressed as mean ± SD.

Slower fluorescence decay and an increase in the contribution of the slow component, generally, reflect a higher reduction of the PSII Q_A_ acceptor. It is clearly seen in the presence of DCMU, an inhibitor of electron transport. The fact of slower fluorescence decay in *PC Mut4* agrees well with the increase in the concentration of non-Q_B_-reducing centers of PSII in cells of this mutant strain.

Reflectance changes at 820 nm (MR) are assigned to the redox transitions of P_700_, a primary electron donor in PSI, and plastocyanin, an electron donor for P_700_^+^. For the simplicity, further in the text we refer MR signal to P_700_. In the dark adapted state, P_700_ is in the neutral state and the reflectance at 820 nm is high. After the onset of illumination MR value rapidly declines, reaching a dip at about 10 ms, followed by the slow recovery to the initial level. These changes reflect the rapid oxidation of P_700_ by the acceptor side of PSI, and the subsequent reduction of P_700_^+^ by electrons arriving from PSII (Figure 4B) shows MR transients normalized to the initial value at 20 μs (MR/MR_0_) recorded simultaneously with the OJIP curves described above.

In *PC Mut2* mutant strain, the initial oxidation, as well as reduction of P_700_ was similar to those in the wild type. In *PC Mut4* mutant strain, P_700_ oxidation was reduced, indicating an inhibition of electron transport via PSI.

Both DF and PF emission originates from radiative deactivation of the singlet excited state of antennae Chl in PSII (Goltsev et al. [2009]). In the case of PF, excited states are created mainly through the absorption of photons by photosynthetic pigments, whereas DF emission is coupled to excited states formed as a result of the backward reactions of electron transport. In order to separate a low yield DF emission from high yield PF signal, DF is recorded in the dark after the excitation pulse offset. DF intensity is proportional to the rates of the corresponding recombination reactions which are strongly affected by the energization of a thylakoid membrane (Wraight and Crofts [1971]). Thus, changes of DF yield during the induction period depend on the formation of electrical and proton differences across the membrane. Fast and slow phases of the DF induction kinetics recorded with M-PEA in plant leaves have recently been analyzed (Goltsev et al. [2009]; Oukarroum et al. [2013]). It is assumed that the fast component (I_1_) (~10-100 ms) reflects transient formation of the electrical gradient across the membrane, likely, due to generation of charge pairs in PSI (P_700_^+^[Fe-S]^−^). The presence of the second DF peak, I_2_, in the seconds range, is associated with the light induced formation of the proton transmembrane gradient (∆pH) that increases the constant of rate of emission transitions in the RC of PSII.

Peaks on DF curve at 20–50 ms, and 1 s were reduced in mutant strains, indicating a decrease in the formation of electrical and proton potential differences across the thylakoid membrane. However, the fast component of DF was reduced to a greater extent in *PC Mut4* strain. The slow phase of the DF induction kinetics (∆pH) was reduced more significantly in *PC Mut2*, compared to the wild type. The last fact agrees with the reduced non-photochemical quenching *q*_E_ = (*F*_*M*_ – *F*_*6s*_)/*F*_*V*_ in *PC Mut2* (Table 1).

These data were confirmed by measurement of fluorescence parameters in algae with a Water-PAM pulse fluorometer. The relative rate of the noncyclic electron transport (*ETR*), calculated as *rETR = Y* × *E*_*i*_ *× 0.5*, and non-photochemical fluorescence quenching *NPQ = (F*_*M*_*- F*_*M*_*’)/ F*_*M*_*’* by electrochemical gradient of protons were reduced in mutants (Table 3).

**Table 3:** **Changes of fluorescence parameters of wild type and mutant strains of** ***Parachlorella kessleri*** **cells**

Fluorescence parameters	Wild type*	PC Mut2*	PC Mut4*
*F*_*V*_ */F*_*M*_	0.58 ± 0.01	0.5 ± 0.01	0.45 ± 0.02
*NPQ*	0.179 ± 0.02	0.133 ± 0.003	0.083 ± 0.004
*rETR*_*max*,_ (r.u.)	15.5 ± 1.3	12.06 ± 0.4	8.3 ± 1.4

*Figures are expressed as mean ± SD.

*F*_*V*_*/F*_*M*_ – samples in darkness, *rETR*_*max*_ – maximum relative rate of electron transport and *NPQ = (F*_*M*_*– F*_*M*_*’)/F*_*M*_*’* – non-photochemical fluorescence quenching at illumination 800 μmol photons m^−2^ s^-1^.

Thus, simultaneous registration of the kinetics of prompt, delayed fluorescence and redox state of P_700_ made it possible to monitor the individual reactions of accumulation of reduced carriers between the photosystems and kinetics of electrochemical proton gradient on a thylakoid membrane of mutant strains *P. kessleri*.

It is known that the constant fluorescence level *F*_*O*_ highly correlates with the total content of pigments in the photosynthetic apparatus of algal cells that harvest light energy (Matorin *et al.*[2004]). The chlorophyll *a* and *b* content in the wild type were 53.3 ± 5.7, 30.2 ± 2.4 mg/l respectively while in the *PCMut2* were 48.3 ± 2.3, 25.2 ± 2.3 mg/l respectively and in the *PCMut4* were 33.3 ± 3.2, 10.2 ± 3.6 mg/l respectively. Fluorescence parameters are used long ago in hydrobiology and oceanology for an assessment of the concentration of microalgae in water and their photosynthetic activity. There is a large number various the submersible fluorometers of different firms for these purposes. Researchers have therefore been trying to find a method of determining the chlorophyll *a* concentration from *in situ* fluorescence measurements. These would cover not only fluorescence induced naturally by sunlight but also that induced by artificial light sources. Measurements of the latter are either contact measurements carried out *in situ* with submersible fluorometers or remote methods using lidars (Pogosyan and Matorin [2005]).

## Conclusion

Microalgae, which use sun energy for synthesis of organic matter, are the main energy source in aquatic systems and serve as the food for other organisms. The linear chain of photosynthetic electron transport comprises two sequentially operating photosystems (PSII and PSI) interconnected through intermediary quinone pool of electron carriers. According to the Z-scheme of electron-transport chain (ETC.), activities of each photosystem affect the redox state of the other system. Such a relation between PSII and PSI is evident from chlorophyll fluorescence, whose level depends on the redox state of the quinone acceptor Q_A_, as well as from the redox state of chlorophyll P_700_, the primary electron donor of PSI. The photoreaction of PSII reduces Q_A_, thus raising chlorophyll fluorescence and the extent of P_700_ reduction, while the PSI reaction converts P_700_ into its oxidized form (P_700_^+^), which promotes Q_A_ oxidation and fluorescence lowering. Simultaneous chlorophyll fluorescence and 820 nm transmission measurements by using М-РЕА-2 allow evaluating the functional state of PSII and PSI. Analysis of fluorescence induction curves demonstrated inhibition of electron transport in PSII and an increase fraction of non-Q_B_-reducing centers. This manifested itself also in an increase of the slow component in decay curve of fluorescence with half-time 2 ns in *PC Mut4*. In addition, analysis of induction curves of the delayed fluorescence and a decrease of NPQ revealed a decrease in the energization of photosynthetic membranes in mutant cells. Our study showed that the most sensitive parameters are induction curves of prompt and delayed fluorescence in mutant algal cells. Selected parameters are indeed viable tools for diagnostic alga state in biotechnological studies during obtaining mutant forms.

## Authors’ original submitted files for images

Below are the links to the authors’ original submitted files for images. Authors’ original file for figure 1 Authors’ original file for figure 2 Authors’ original file for figure 3 Authors’ original file for figure 4 Authors’ original file for figure 5 Authors’ original file for figure 6 Authors’ original file for figure 7 Authors’ original file for figure 8

## Abbreviations

PSII: Photosystem II
RC: Reaction center
Q_A_: Primary
Q_B_: Secondary quinone acceptors of electron
PQ: Plastoquinone
O: J, I, P, Intermediate levels of the light-induced fluorescence curve
DF: Delayed Chl fluorescence
Р_700_: Pigment of the RC of PSI
