# Supplementary figures and images for: Differentiation between two strains of microalga Parachlorella kessleri using modern spectroscopic method
Source: Bot Stud. 2014 Jul 12;55:53. doi: 10.1186/s40529-014-0053-7 (PMC5430349; doi:10.1186/s40529-014-0053-7)

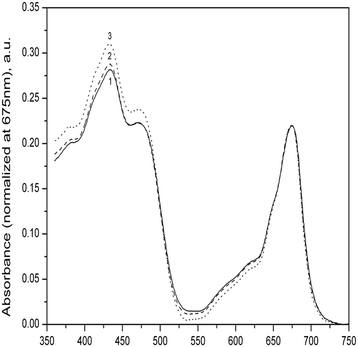

Supplement: Supplementary file 1 — Authors’ original file for figure 1 [file 40529_2014_9053_MOESM1_ESM.gif]

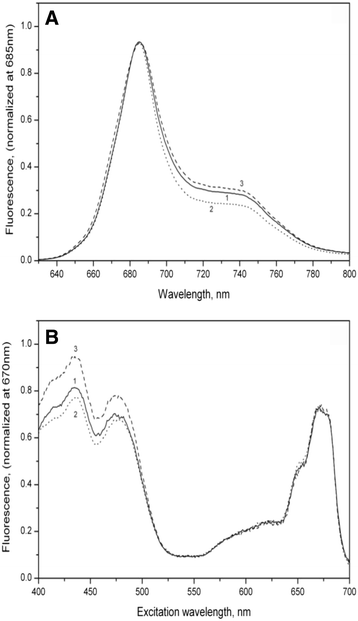

Supplement: Supplementary file 2 — Authors’ original file for figure 2 [file 40529_2014_9053_MOESM2_ESM.gif]

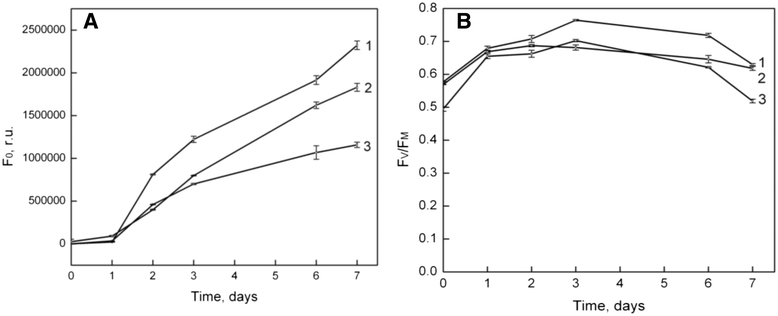

Supplement: Supplementary file 3 — Authors’ original file for figure 3 [file 40529_2014_9053_MOESM3_ESM.gif]

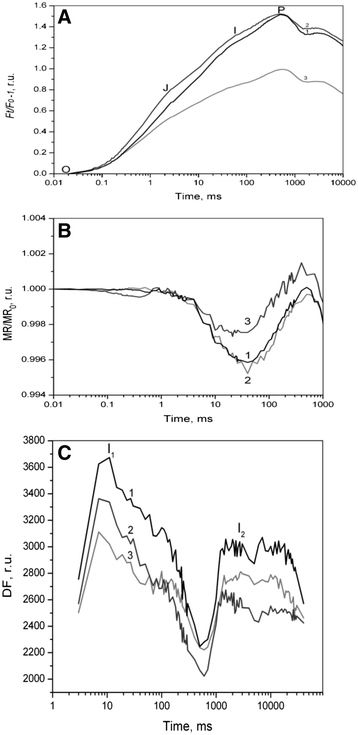

Supplement: Supplementary file 4 — Authors’ original file for figure 4 [file 40529_2014_9053_MOESM4_ESM.gif]

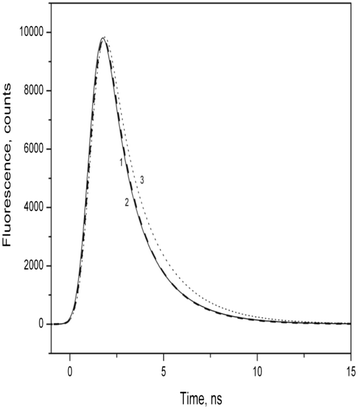

Supplement: Supplementary file 5 — Authors’ original file for figure 5 [file 40529_2014_9053_MOESM5_ESM.gif]
