# Supplementary material for: Differentiation between two strains of microalga Parachlorella kessleri using modern spectroscopic method
Source: Bot Stud. 2014 Jul 12;55:53. doi: 10.1186/s40529-014-0053-7 (PMC5430349; doi:10.1186/s40529-014-0053-7)
Supplement: Supplementary file 6 — Authors’ original file for figure 6 [file 40529_2014_9053_MOESM6_ESM.doc]

**Table 1.**Parameters of OJIP kinetics of fluorescence induction curve measured in wildtype and two mutant strains of *Parachlorella kessleri* cells. The kinetic parameters of fluorescence induction were measured on M-PEA-2 fluorometer under actinic illumination of 1300 μE/(m^2^ s)

| JIP test parametrs | | Wild type *PC* | *PCMut2* | *PCMut4* |
| --- | --- | --- | --- | --- |
| *F_V_/F_M_* | Maximum quantum yield of charge separation in PSII | 0.610 | 0.607 | 0.506 |
| *V_J_* | Relative amplitude of the O-J phase | 0.38 | 0.47 | 0.48 |
| *V_I_* | Relative amplitude of the J-I phase | 0.96 | 0.96 | 0.94 |
| *M_O_* | Initial slope of the O-J fluorescence phase | 0.60 | 0.73 | 0.87 |
| *S_M_* | Area between the fluorescence kinetic curve (O–J–I–P) and the level of Fm normalized against the  *F_V_* value | 40.97 | 38.72 | 30.06 |
| *ABS/RC* | Average value of absorbed photon flows in PSII RC (of apparent size of the active antenna in PSII) | 2.57 | 2.85 | 3.51 |
| *q_E_* | Capacity for pH-induced non-photochemical fluorescence quenching | 0.14 | 0.12 | 0.10 |
| *q_PQ_* | Capacity of the quinone pool for fluorescence quenching | 0.24 | 0.20 | 0.19 |
