# Supplementary material for: Differentiation between two strains of microalga Parachlorella kessleri using modern spectroscopic method
Source: Bot Stud. 2014 Jul 12;55:53. doi: 10.1186/s40529-014-0053-7 (PMC5430349; doi:10.1186/s40529-014-0053-7)
Supplement: Supplementary file 7 — Authors’ original file for figure 7 [file 40529_2014_9053_MOESM7_ESM.doc]

**Table 2.** Change of amplitude and duration of fast (A1, τ_1_) and slow (A2, τ_2_) components of fluorescence decay kinetics measured in wildtype and mutant strains of *Parachlorella kessleri* cells .

| 1. Fluorescence parameters | 1. A1 (%) | 1. A2 (%) | 1. τ_1_ (ns) | 1. τ_2_ (ns) |
| --- | --- | --- | --- | --- |
| 1. wildtype | 1. 40 | 1. 60 | 1. 1.0+-0.13 | 1. 2.24+-0.03 |
| 1. *PCMut2* | 1. 37 | 1. 63 | 1. 0.97+-0.14 | 1. 2.18+-0.03 |
| 1. *PCMut4* | 1. 28 | 1. 72 | 1. 1.09+-0.23 | 1. 2.47+-0.04 |
