# Supplementary material for: Differentiation between two strains of microalga Parachlorella kessleri using modern spectroscopic method
Source: Bot Stud. 2014 Jul 12;55:53. doi: 10.1186/s40529-014-0053-7 (PMC5430349; doi:10.1186/s40529-014-0053-7)
Supplement: Supplementary file 8 — Authors’ original file for figure 8 [file 40529_2014_9053_MOESM8_ESM.doc]

**Table 3.** Changes in fluorescence parameters - *F_V_/F_M_* – samples in darkness, *rETR_max_* - maximum relative rate of electron transport and *NPQ=(F_M_ - F_M_')/ F_M_ '*- nonphotochemical fluorescence quenching at illumination 800 µE/(m^2^ s) in wild type and mutant strains *Parachlorella kessleri* cells.

| 1. Fluorescence parameters | 1. wildtype | 1. *PCMut2* | 1. *PCMut4* |
| --- | --- | --- | --- |
| 1. *F_V_/F_M_* | 1. 0.58 | 1. 0.5 | 1. 0.45 |
| 1. *NPQ* (800 μE/м^2^с) | 1. 0.179 | 1. 0.133 | 1. 0.083 |
| 1. *rETR_max_*, (r.u.) | 1. 15.5 | 1. 12.06 | 1. 8.3 |
